# Diabetic retinopathy (DR): management and referral

**Published:** 2011-09

This diabetic retinopathy (DR) grading system is based on the International Council of Ophthalmology's diabetic retinopathy and diabetic macular oedema disease severity scales (see Useful Resources on page 19). At whatever level you work, you must **encourage everyone with diabetes to manage their blood sugar and blood pressure**. Refer them to available services for help if they are not sure how to do this, or if their control is poor.

**Figure:**
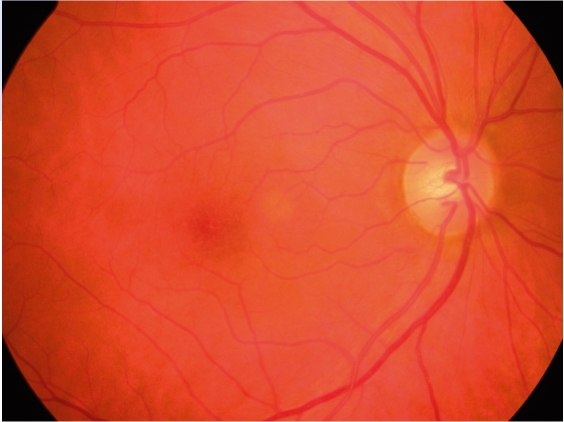
No abnormalities

**Figure:**
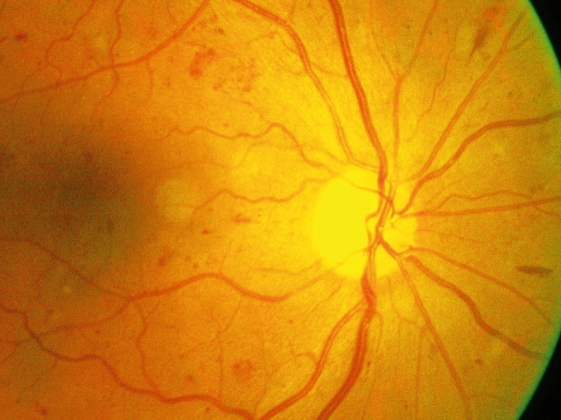
**Microaneurysms (small, round ‘dots’) and haemorrhages (larger, uneven ‘blots’)**. An example of moderate non-proliferative DR

**Figure:**
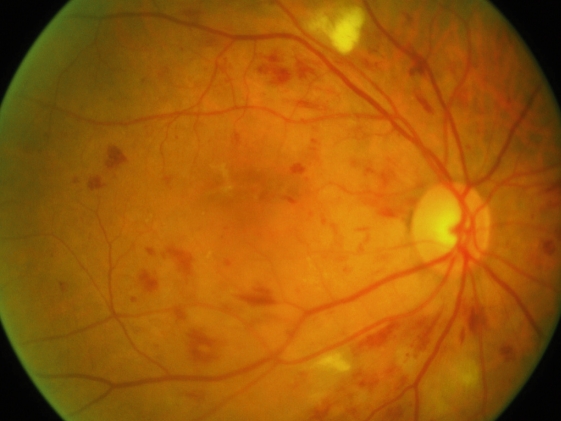
**Cotton wool spots (white), haemorrhages, and microaneurysms**. An example of severe non-proliferative DR

**Table 1:** Expected increase in number of people with diabetes: 2010-2030

Diabetic retinopathy stage	Clinical signs	What to do (screening/primary eye care)	What to do (retinal clinic)	What you could say to your patients
**No diabetic retinopathy**	No abnormalities	Encourage patient to come again in 12 months	Review in 12 months	Diabetes can affect the inside of your eyes at any time. It is important that you come back in twelve months so we can examine you again. This will help to prevent you losing vision or going blind.
**Mild nonproliferative diabetic retinopathy**	Microaneurysms only	Encourage patient to come again in 12 months	Review in 12 months	Your diabetes is affecting your eyes. At the moment your vision is good, but we must check your eyes in 12 months' time to see if these changes are getting worse. If the damage becomes severe, we will need to treat your eyes to stop the diabetes affecting your sight.
**Moderate nonproliferative diabetic retinopathy**	More than just microaneurysms but less than severe non-proliferative retinopathy	Encourage patient to come again in 6-12 months	Review in 6-12 months	Your diabetes is damaging your eyes. At the moment your vision is good, but we must check your eyes in six months' time as it is likely that these changes will get worse. If the damage becomes severe, we will need to treat your eyes to stop the diabetes affecting your sight. Unless you are treated promptly, you risk losing vision or going blind.
**Severe nonproliferative diabetic retinopathy**	More than 20 haemorrhages in each quadrant; orvenous beading in two quadrants; or intraretinal microvascular abnormalities (IRMA)	Refer to retinal clinic. All patients with severe non-proliferative DR should be in the care of an ophthalmologist. The patient should be re-examined every six months	Either review in 6 months, or consider pan-retinal laser, if follow-up unreliable	Your diabetes has damaged your eyes quite severely, although your vision is still good. You are likely to need treatment soon to ensure that you don't lose vision or go blind. We must check your eyes in six months' time. However, if you think you may not be able to come then, we may treat your eyes now, so we can be sure you don't lose vision later.
**Proliferative diabetic retinopathy**	Any new vessels at the disc or elsewhere, vitreous/pre-retinal haemorrhage	Urgent referral to retinal clinic	Pan-retinal laser/vitrectomy if vitreous haemorrhage or retinal detachment	Your diabetes has damaged your eyes very severely. Although your vision may be good, you are at great risk of losing your sight over the next year. You need urgent treatment to save your sight. Treatment will not improve your eyesight, but should preserve the vision you have.
**Macular oedema**				
**Macular oedema absent**	No exudates or retinal thickening in posterior pole	Review in 12 months	Review in 12 months	As for “No diabetic retinopathy” above.
**Mild macular oedema**	Exudates or retinal thickening at posterior pole, >1dd from fovea	Review in 6 months	Review in 6 months	Your diabetes is damaging your eyes. At the moment your vision is good, but we must check your eyes in six months' time as it is likely that these changes will get worse. If the damage becomes severe, we will need to treat your eyes to stop the diabetes affecting your sight. Unless you are treated promptly, you risk losing vision or going blind.
**Moderate macular oedema**	Exudates or retinal thickening at posterior pole, ldd or less from fovea, but not affecting fovea	Refer to retinal clinic. Encourage patient to manage their blood sugar and blood pressure, and refer them to available services for help if they are not sure how to do this	Laser treatment if clinically significant macular oedema (CSMO) Review in 6 months if no CSMO	Your diabetes has damaged your eyes severely. Although your vision may be good at present, it is likely to get worse over the next year or two. You need laser treatment to stop your sight deteriorating. The treatment will not improve your eyesight, but should preserve the vision you have.
**Severe macular oedema**	Exudates or retinal thickening affecting centre of fovea	Refer to retinal clinic	Laser treatment or intravitreal injections of anti-VEGF drugs	You have probably noticed your eyesight has got worse. This is because your diabetes has damaged your eyes very severely. You need urgent treatment to prevent further loss of vision. The treatment may not improve your eyesight, but if you are not treated, your vision will get worse and you may even become blind.

If you cannot see the retina due to cataract or vitreous haemorrhage, refer to an ophthalmologist for cataract surgery or a retinal sugeon for vitrectomy.

**Figure:**
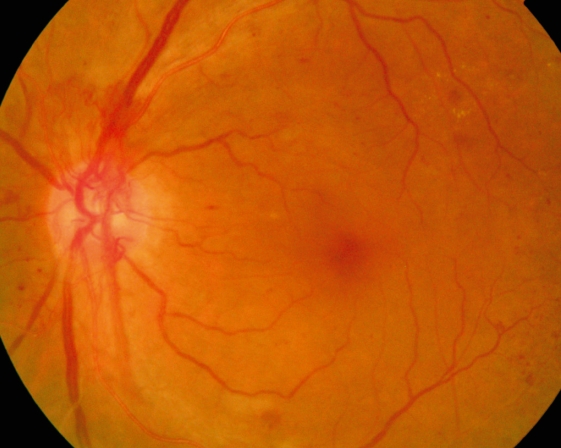
**New vessels on the optic disc**. An example of proliferative DR

**Figure:**
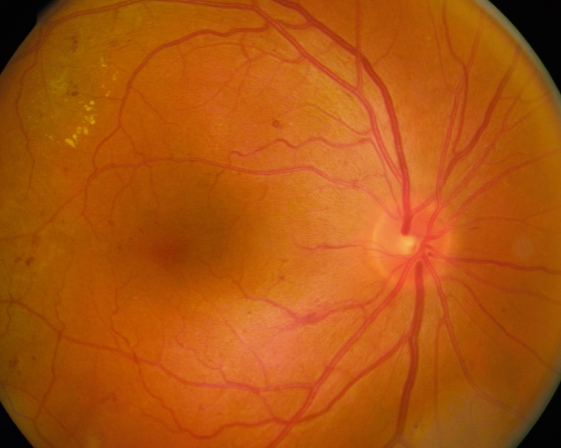
**Exudates (bright yellow)**. An example of mild macular oedema

**Figure:**
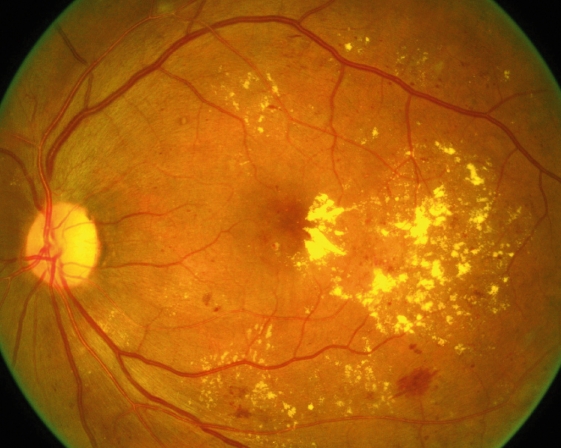
**Exudates**. An example of severe macular oedema

